# Compact neural network algorithm for electrocardiogram classification

**DOI:** 10.1007/s13246-026-01751-1

**Published:** 2026-06-11

**Authors:** Christian M. Frausto-Avila, Juan P. Manriquez-Amavizca, Ana K. S. Rocha-Robledo, Mario A. Quiroz-Juarez, Alfred B. U’Ren

**Affiliations:** 1 Centro de Física Aplicada y Tecnología Avanzada, Universidad Nacional Autónoma de México, Boulevard Juriquilla 3001, 76230 Querétaro, Querétaro México; 2 Instituto de Ciencias Nucleares, Universidad Nacional Autónoma de México, 04510 Querétaro, Querétaro México; 3 Instituto Tecnológico y de Estudios Superiores de Monterrey, Universidad Nacional Autónoma de México, Epigmenio González 500, Fracc, San Pablo, 76130 Querétaro, Querétaro México

**Keywords:** Machine learning, Neural networks, Arrhythmia classification, ECG

## Abstract

In this paper, we present a powerful, compact electrocardiogram (ECG) classification algorithm for cardiac arrhythmia diagnosis that addresses the current reliance on deep learning and convolutional neural networks (CNNs) in ECG analysis. This work aims to reduce the demand for deep learning, which often requires extensive computational resources and large labeled datasets. Our approach introduces an artificial neural network (ANN) with a simple architecture combined with a compact, interpretable feature-engineering pipeline. A key contribution of this work is the incorporation of 17 engineered features that enable the extraction of critical patterns from raw ECG signals. By integrating mathematical transformations, signal processing methods, and data extraction algorithms, our model captures the morphological and physiological characteristics of ECG signals with high efficiency, without requiring deep learning. Our method demonstrates a similar performance to other state-of-the-art models in classifying five rhythm classes—normal sinus rhythm, sinus bradycardia, sinus tachycardia, ventricular flutter, and atrial fibrillation. Our algorithm achieved an accuracy of $96.48\% \pm 2.50\%$ on the MIT-BIH and St. Petersburg INCART arrhythmia databases, with a Cohen’s kappa of *0.9546 ± 0.0321* and a Matthews correlation coefficient of *0.9557 ± 0.0313*. The compactness of the model and its low inference latency on a standard CPU suggest that the approach is a promising candidate for deployment on resource-constrained platforms.

## Introduction

Arrhythmic diseases are defined as irregularities in the normal functioning of the electrical impulses that are responsible for regulating the heartbeat. Arrhythmias can manifest in various forms and trigger multiple health issues. Some arrhythmias are benign, while others may represent life-threatening risks. The World Health Organization (WHO) indicates that cardiovascular diseases remain the leading cause of death worldwide. They account for 31% of all global deaths [1, 2]. Consequently, the study and development of methodologies for the detection of arrhythmias through technological devices are crucial for maintaining public health and reducing the risk of fatal cardiac disorders. The electrocardiogram (ECG) is a widely employed tool that plays an essential role in this important task. First introduced in 1902 by Willem Einthoven [3], ECG reflects the macroscopic electrical activity of the heart, generating a visual record of this activity as a function of time. This electrical activity governs the contractions and relaxations of the heart muscle that occur with each heartbeat. Due to its non-invasive nature, ECG recordings are currently the most widely used method for evaluating a patient’s cardiovascular health and are considered a universal standard in clinical practice and cardiological studies. Approximately, 300 million ECGs are recorded every year [4]. Importantly, the relevance of ECGs has reached the academic world. Significant research efforts have been dedicated to modeling and understanding ECG signals, in order to study and comprehend the macroscopic electrical activity of the heart [5–9].

**Fig. 1:**
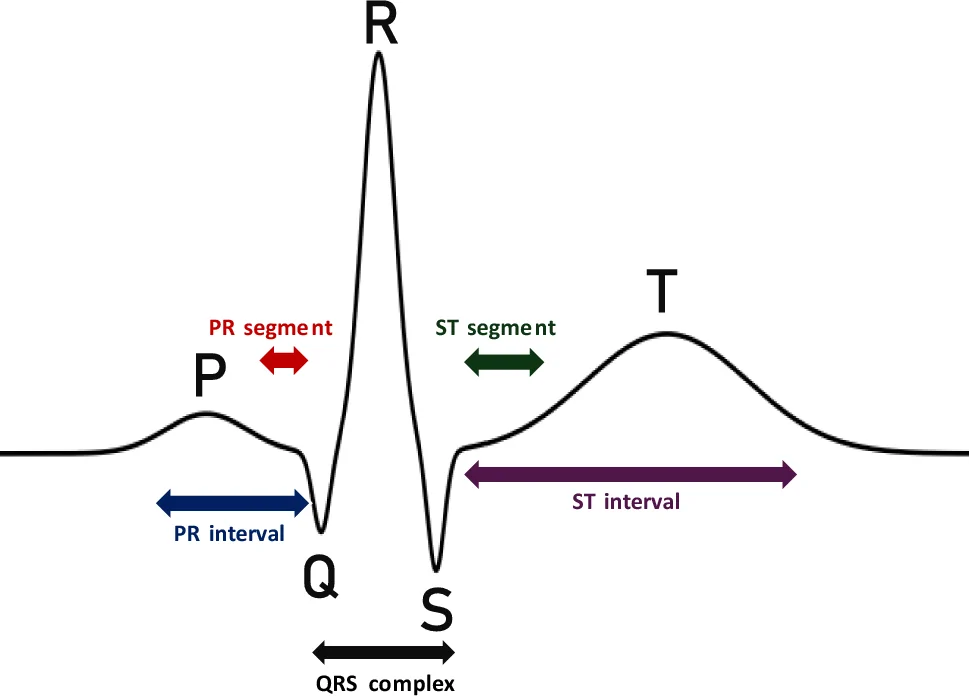
Typical PQRST pattern present in a healthy body

Under normal conditions, the ECG signal follows a characteristic PQRST waveform, which represents the sequential depolarization and repolarization events regulating heart function. Figure 1 illustrates the peaks and troughs of the ECG waveform, labeled P, Q, R, S, and T. This pattern is standard in healthy individuals, and deviations from it may indicate cardiac conditions such as arrhythmias. ECG is a reliable tool for detecting abnormalities in the PQRST pattern and diagnosing various cardiac disorders, including tachycardia (heart rate >100 BPM), bradycardia (<60 BPM), hypertension, hypotension, and myocardial infarction [10, 11]. It is also effective in identifying other arrhythmias such as atrial and ventricular fibrillation, congenital heart defects, heart block, coronary artery disease, pericarditis, cardiomyopathy, electrolyte imbalances, and rheumatic heart disease. However, ECG-based diagnosis has limitations. Interpretation often relies on the clinician’s expertise, and factors like noise or similarities between arrhythmic disorders can complicate the analysis. Moreover, the same arrhythmia can manifest with different symptoms and rhythm abnormalities, posing a challenge even for experienced cardiologists. Given these challenges, developing techniques to enhance and simplify cardiac data analysis is essential.

Notably, machine learning (ML) has emerged as a powerful tool for identifying arrhythmic disorders from clinical ECG signals. Its ability to recognize intricate patterns in complex data has led to significant advancements across various scientific disciplines [12–15]. In ECG classification tasks, convolutional neural networks (CNNs) have been employed to enhance classification accuracy. For instance, Ref. [16] proposed a CNN that automates feature extraction, further streamlining the analysis process. Similarly, Jun et al. [17] developed a two-dimensional CNN that achieved an impressive accuracy of 99.05%. Wang et al. [18] introduced another CNN architecture that incorporates the continuous wavelet transform (CWT) as an input feature, achieving a classification accuracy of 98.74%. Note that these models eliminate the need for manual feature engineering and are inherently more robust to noise. However, deep learning approaches require large datasets and significant computational resources for training, posing challenges for real-world implementation.

Alternatively, advanced feature engineering techniques have been proposed to extract relevant information and intricate patterns from raw ECG signals. For instance, Sadhukhan et al. [19] applied a discrete Fourier transform (DFT) to the ECG signal as an engineered feature for an ML model, achieving a performance of 95.6%. However, this approach was limited to the detection of myocardial infarction. Likewise, Zeng et al. [20] implemented the Shannon energy envelope as an extracted feature, reaching an accuracy of 99.21%, though it was also restricted to detecting a single cardiac disorder. Other methods for ECG signal classification involve approaches based on support vector machines (SVM), decision tree algorithms, and K-Nearest Neighbors (KNN) classifiers. Rabee and Barhumi [21] developed an SVM-based approach for the recognition of 14 distinct heartbeats from the MIT-BIH arrhythmia database. The authors applied a wavelet transform to the signal for feature extraction. Their classifier achieved a 99.2% accuracy. Kumari et al. [22] engineered a decision tree to classify six types of heartbeats, including normal rhythm and five arrhythmic types. The authors obtained an accuracy of 94.5%, after extracting 17 morphological features from the signals.

Mohebbanaaz and Padma [23] developed a KNN-based approach and achieved a classification of 99.03% using the MIT-BIH database with 6 types of ECG beats. Normal sinus rhythm (NSR), left bundle branch block (LBBB), right bundle branch block (RBBB), premature ventricular contraction (PVC), atrial premature beat (APB), and paced beat (PAB). However this approach required a boosting algorithm for hyperparameter optimization that increased the complexity of the whole methodology, without which the accuracy drop to 94.5%. Nevertheless, these methods demonstrate the importance of feature engineering for ECG signal classification, which plays a crucial role in early detection and diagnosis of cardiovascular diseases, requiring efficient and accurate machine learning models. This approach has allowed us to engineer a more robust classification algorithm.

**Fig. 2:**
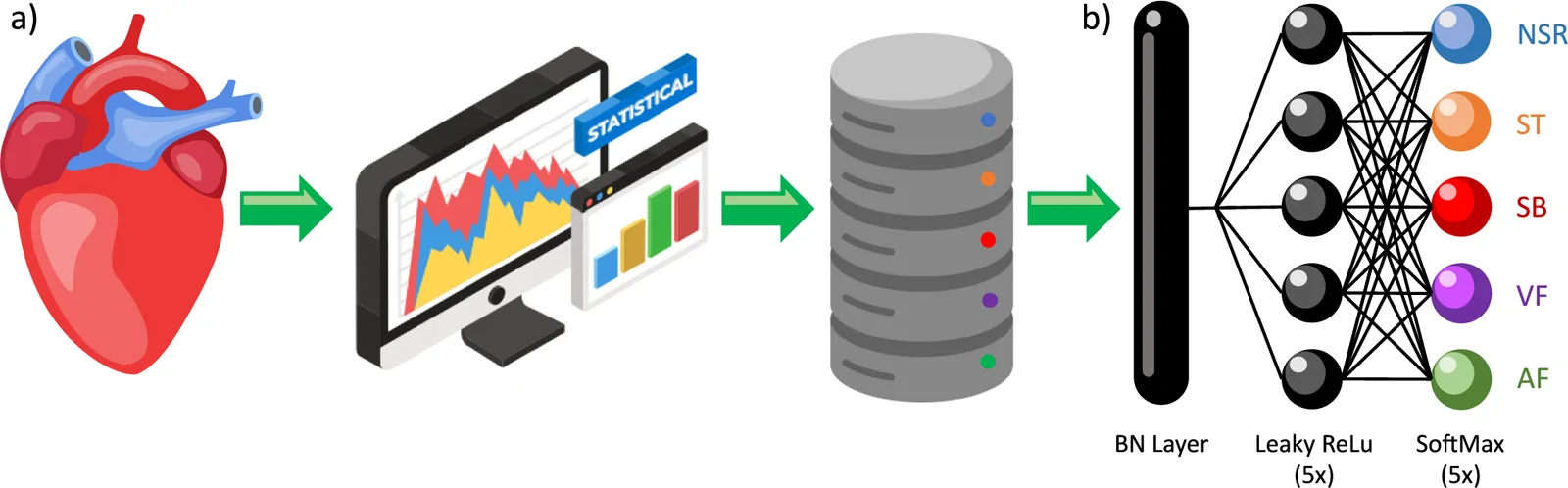
General scheme of our approach. **a** Real recordings obtained from patients at both the Beth Israel Deaconess Medical Center and St. Petersburg Institute of Cardiological Technics, Feature engineering of the ECG recording and the database creation from Normal Sinus Rhythm, SB to Sinus Bradycardia, ST to Sinus Tachycardia, VF to Ventricular Flutter, and AF to Atrial Fibrillation and **b** Neural Network architecture

Our study has been significantly motivated by recent advancements in deep learning. These studies have demonstrated large potential in medical diagnostics using machine learning algorithms and data extraction techniques. One particularly influential approach is triplet representation learning, as explored by [24]. This technology is designed to improve feature engineering and data extraction processes through the implementation of the SimTrip representation learning model. This methodology has been successfully implemented for medical image classification and it demonstrated its effectiveness in learning discriminative features for high-dimensional and complex medical data. Inspired by this scientific advancement, we have integrated advanced feature extraction techniques in our ECG classification algorithm study to improve robustness, generalizability and a lower computational complexity. By carefully implementing data extraction principles, we aim to improve the model’s ability to distinguish subtle variations in ECG signals by capturing important morphological and statistical features. This approach has ultimately allowed us to engineer a more robust classification algorithm. Additionally, our work was influenced by [25], which highlights how generative adversarial networks (GANs) can generate synthetic lung cancer images and extract key features for their classification. The study incorporates CNNs to extract intricate morphological information from complex medical data. CNNs have extensively demonstrated how deep learning models can be employed for feature extraction in biomedical engineering applications and diagnostic tools. As a result, we focus on developing a streamlined approach for feature extraction using mathematical transformations and data extraction techniques like Principal Component Analysis (PCA). Rather than relying solely on deep learning networks, our method integrates feature engineering with a compact neural network architectures to maximize the efficiency of ECG classification while maintaining model simplicity. Furthermore, our work has been motivated by recent advancements in large foundation models for medical imaging, as explored by [26]. This study has demonstrated the capacity of efficient neural networks in processing complex medical data. These models exhibit the potential of feature-efficient architectures in handling diverse and high-dimensional medical data with complex and intricate patterns hidden within them.

In this study, we propose a compact neural network-based classification framework for the detection of four arrhythmic disorders from real clinical electrocardiogram records. Unlike previous feature-based ECG classifiers that rely either on a single family of descriptors (e.g., wavelet coefficients, Fourier harmonics, or morphological intervals) or on heavyweight ensemble pipelines with boosting, our contribution is threefold. First, we construct a heterogeneous 17-dimensional descriptor that fuses statistical, morphological, spectral, analytic-signal, and PCA-based information extracted from the same raw signal, thereby exposing complementary representations of the cardiac dynamics to the classifier. Second, we deliberately constrain the network to a single hidden layer of five neurons, which keeps the total number of trainable parameters at 154 (at least three orders of magnitude smaller than the CNN, Self-ONN, and wavelet-ANN baselines summarized in Table 2.) Third, we incorporate class weighting during training to compensate for the imbalance of the dataset without resorting to synthetic data augmentation. The resulting low-dimensional model attains an overall classification accuracy of *96.48%*, achieving a favorable balance between computational efficiency and diagnostic performance, and supports deployment in resource-constrained mobile healthcare scenarios.

## Methodology

The goal of our work is to classify arrhythmic diseases from real ECG signal data through the implementation of a minimally complex neural network algorithm. To achieve this, we first built a database of ECG recordings from real clinical records to train our model. Next, we applied a curated combination of feature engineering techniques drawn from prior ECG and signal-processing literature to extract key characteristics and intricate patterns from the ECG signals as shown in Fig. 2. The proposed features served as inputs to the ML model. Finally, the algorithm undergoes hyperparameter optimization to maximize its predictive performance. Figure 2 shows a general scheme of our approach for the ECG classification task.

### Database

Our initial efforts focused on collecting data from real ECG clinical records. This work focuses on the classification of four types of arrhythmias plus normal sinus rhythm as a reference class. The dataset is composed of ECG recordings from the open MIT-BIH Arrhythmia Database, which is available at the PhysioNet platform and contains data from 47 subjects [16]. These recordings were initially obtained from patients at the Beth Israel Deaconess Medical Center, located at Boston. The recordings were part of a series of cardiology experiments conducted between 1975 and 1979 [27]. Moreover, the database includes 23 randomly selected recordings from a total of 4000 24-hour ambulatory ECG recordings collected at the same institution. These additional recordings contain rare and clinically significant arrhythmic diseases for academic and research purposes. For this work, we used all available recordings. All samples have a duration of 30 min. They were originally recorded at 360 Hz, with 11-bit resolution over a 10 mV dynamic range. In order to increase the size of our dataset and diversify the sources of the ECG signals used to train the algorithm, we incorporated ECG recordings from the open St. Petersburg INCART 12-lead Arrhythmia Database, which is also available at PhysioNet. The source is composed of 75 recordings from 32 patients collected at the St. Petersburg Institute of Cardiac Technics [28]. These recordings were originally sampled at 257 Hz. Each recording contains 12 different signals that correspond to a different electrode placement. Note that in this study, they used the standard Lead I, which consists of placing the positive electrode on the left arm and the negative electrode on the right arm.

**Fig. 3:**
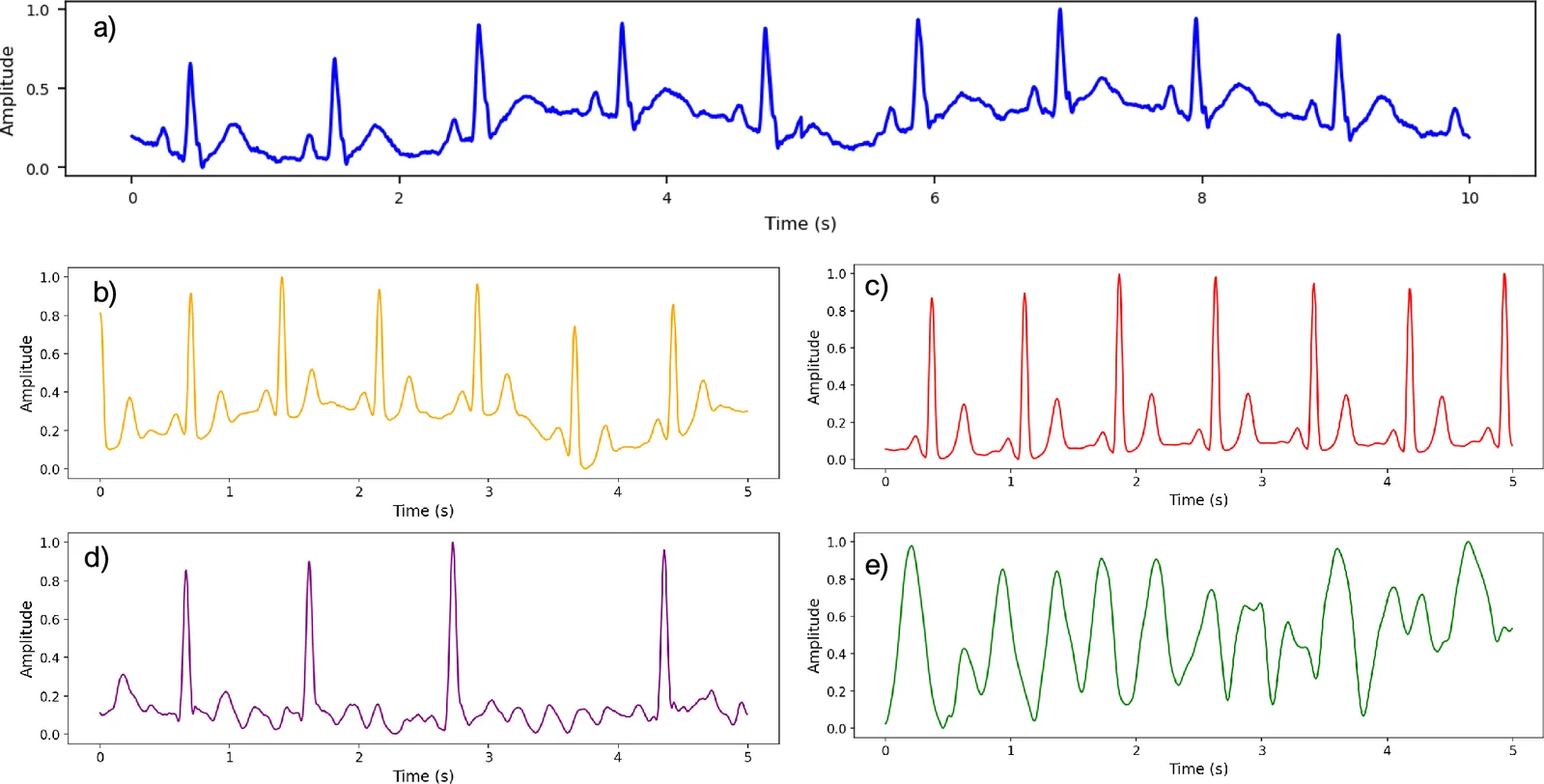
ECG recording samples of 5 s of **a** Normal Sinus Rhythm, **b** Sinus Bradycardia, **c** Sinus Tachycardia, **d** Ventricular Flutter, and **e** Atrial Fibrillation

Our final database contained 97 samples of Normal Sinus Rhythm (NSR), which serves as a standard reference of a healthy cardiac behavior. A sample from this pattern is displayed in Fig. 3a). Additionally, our database includes 100 samples of sinus bradycardia, 100 samples of sinus tachycardia, 24 samples of ventricular flutter, and 105 samples of atrial fibrillation. Each sample was 5 s long and displayed a continuous arrhythmic pattern. A sample from each arrhythmia is displayed in figures 3b), 3c), 3d) and 3e).

### Signal preprocessing

To standardize the data and ensure compatibility of all samples with the training phase, we performed thorough signal preprocessing. The MIT-BIH Arrhythmia Database ECG signals were processed using a downsampling algorithm to reduce the sampling frequency to 257 Hz, matching the sampling frequency of the St. Petersburg INCART Database.

Importantly, the signals were divided into 5-s segments. This process generated a dataset with 1285 features. Each feature represents the electrical signal, measured in millivolts (mV). Subsequently, we applied a moving average algorithm to all samples with a window size of 12 samples to smooth the signals. This approach significantly reduced noise without compromising substantial information from the raw data. This final step led to a dataset with 1273 features. Finally, the data was normalized to a range of 0 to 1 using the min-max scaling algorithm.

### Model and architecture

ML can be defined as a collection of algorithms capable of identifying intricate patterns within data and provide decision-making for specific tasks. As previously remarked, neural networks have gained prominence as a powerful and widely used ML algorithm approach. Neural networks consist of a series of interconnected nodes, or neurons, which perform a transformation. Each neuron computes an output by applying a nonlinear function, known as activation function, to a weighted sum of its inputs [29]. During the training stage, the synaptic weights of each neuron are automatically adjusted through an optimization algorithm that minimizes a loss function. Finally, the performance is evaluated in the testing phase. Increasing the number of neurons in the network leads to higher computational cost, greater algorithmic complexity, and an elevated risk of overfitting. Overfitting is a common issue in supervised machine learning that hinders the creation of generalized models [30]. It occurs when the model becomes overly specialized in the training data. Consequently, the algorithm performs poorly when evaluated with unseen data, resulting in poor generalization.

Our approach employs a single-hidden-layer architecture, substantially reducing the number of neurons in the network to lower computational costs and facilitate deployment in real-world applications. As a crucial part of our approach, we incorporated advanced feature engineering and signal analysis techniques to extract the maximum amount of relevant information from the raw ECG data. The combination of a simplified architecture and sophisticated data processing enables efficient and effective classification while minimizing resource requirements.

The input layer is configured to match the dimensionality of the feature vector, comprising seventeen characteristics. A batch normalization (BN) layer is then applied directly to its output to stabilize the learning process. The normalized data are subsequently passed to a hidden layer containing five neurons, each employing a "leaky_relu" activation function. The output layer consists of five neurons (one per target class), each using a softmax activation to produce class probabilities. This architecture ensures a powerful and stable model while maintaining simplicity.

The proposed model was evaluated using stratified 10-fold cross-validation, repeated 5 times with independent random seeds, yielding 50 disjoint test folds in total. Stratification preserves the class distribution in every fold, which is essential given the imbalance of the Ventricular Flutter class. Model hyperparameters were fixed prior to this evaluation on the basis of the sensitivity analysis reported in Hyperparameter sensitivity and feature ablation section; no hyperparameter tuning was performed against the test folds. To prevent information leakage from the feature pipeline, both the PCA basis and the batch-normalization statistics were estimated solely on the training fold of each cross-validation iteration and then applied unchanged to the corresponding test fold. The Adam optimizer [31] was used with a learning rate of $\alpha = 1\times 10^{-3}$, a batch size of 20, and 220 training epochs.

### Hyperparameter sensitivity and feature ablation

The number of hidden units was selected via a sensitivity analysis over $\{2, 3, 5, 8, 12, 20, 40\}$ neurons under the stratified-CV protocol described above. Mean test accuracy plateaued at five units, with larger configurations yielding statistically indistinguishable performance (overlapping one-standard-deviation intervals) at the cost of additional parameters. We therefore selected the smallest configuration on the plateau, in line with the compactness goal of this work. A complementary feature-ablation study, in which each feature family is removed in turn, is reported in Fig. 5 and confirms that the peak/heart-rate descriptors and the statistical moments dominate the prediction, while the Hilbert-envelope, DFT, and Shannon-entropy families contribute marginally when the others are present.

### Feature engineering

We emphasize that the individual descriptors employed in this work (statistical moments [32, 33], heart-rate and inter-peak features, PCA components [34, 35], DFT-derived statistics [19, 36], Hilbert-envelope descriptors [37, 38], and Shannon entropy [39]) are individually well-established in the ECG literature. The contribution of this work is therefore not the introduction of new descriptors, but the demonstration that a carefully selected, low-dimensional composite of 17 such descriptors is sufficient to drive a single-hidden-layer network with only five neurons to a classification performance comparable to that of substantially deeper convolutional architectures, while preserving interpretability and a low computational footprint.

To optimize the performance of the proposed neural network without increasing its architectural complexity, advanced feature engineering techniques were incorporated. The resulting feature vector comprised 17 key metrics derived from ECG signal data. These included the four classical statistical moments–mean, kurtosis, skewness, and variance [1]–as well as three clinically relevant features: cardiac rhythm, peak amplitude variability, and the average inter-peak interval. Collectively, these widely used features have been shown to be sufficient for classifying ECG signals with acceptable accuracy [4]. In particular, the statistical moments have demonstrated relevance and reliability in capturing morphological characteristics of the data distribution and the underlying signal dynamics [32, 33]. However, to achieve an accuracy exceeding 95%, the proposed algorithm further integrates advanced feature engineering strategies.

Among these strategies, dimensionality reduction plays a crucial role in enhancing model efficiency while preserving essential information. Principal Component Analysis (PCA) is a statistical method that reduces the dimensionality of data. PCA is widely employed in signal studies to condense the data into key components that capture the most significant information and intricate characteristics. Interestingly, PCA has proven to be valuable in ECG analysis and signal processing. It is a particularly effective tool for extracting patterns and identifying physiological abnormalities. [34]. Additionally, researchers have employed PCA for the classification of arrhythmic diseases to reduce data dimensionality while preserving critical features [35]. For this work, we retain the first three principal components, which together explain $72.34\% \pm 0.15\%$ of the variance of the smoothed ECG signal across the training folds of the stratified cross-validation. To prevent information leakage, the PCA basis is fit exclusively on the training fold of each cross-validation iteration and subsequently applied without modification to the corresponding test fold.

Another component of our feature engineering strategy involved applying mathematical transformations to the ECG signals. As demonstrated by Sadhukhan et al. [19], such transformations can extract discriminative features that enhance the interpretability of ECG data and substantially improve classification performance. Accordingly, we applied the Discrete Fourier Transform (DFT) to each ECG signal. The Fourier transform has been widely used in ECG analysis as a feature extraction tool, enabling models to achieve classification accuracies exceeding 97% [36]. The DFT of a sequence $x_0\,\,, x_1\,\,, \dots , x_{N-1\,\,}$ is defined as follows:

1 $$\begin{aligned} X_k = \sum _{n=0}^{N-1} x_n e^{-i 2 \pi \frac{k n}{N}}, \end{aligned}$$

where $X_k$ represents the * k *-th frequency component of the DFT, and $x_n$ are the time-domain samples with $n = 0\,\,, 1\,\,, \dots , N-1\,\,$. Here, * N * is the number of samples in the sequence, and $e^{-i 2\,\, \pi \frac{k n}{N}}$ is a complex exponential function that represents the basis functions of the Fourier transform. In this context, * i * is the imaginary unit ($i = \sqrt{-1\,\,}$), and $k = 0\,\,, 1\,\,, \dots , N-1\,\,$ represents the frequency indices. The purpose of the DFT is to convert the original time-domain signal into its frequency components, which are represented by the complex values $X_k$. These components contain the amplitude and phase information of the signal at different frequencies. After applying the algorithm, the Fourier transformation generated a set of complex numbers that represent the signal’s frequency components. This data was then processed to generate two derived features: skewness and kurtosis.

In addition to the DFT, we applied the Hilbert Transform, a widely used method in ECG signal studies and research. [1, 37]. The purpose of the Hilbert Transform is to generate an enveloping function that captures the essential oscillatory behavior of ECG signals. The envelope generated from the Hilbert Transform is defined as a function of time applied to a a signal * x(τ ) * and is given by the following formula:

2 $$\begin{aligned} H\{x\}(t) =\frac{1}{\pi } \mathcal {\mathcal {P}}\int _{-\infty }^{\infty }\frac{x(\tau )}{t-\tau } d\tau \end{aligned}$$

where $\mathcal {P}$ denotes the principal value of the integral, and * t * and * τ * represent time variables [38]. After applying the transform, we processed the obtained signal to extract the mean, minimum amplitude, and maximum amplitude from the resulting function.

Another key feature in our methodology is entropy, a metric quantifying a signal’s unpredictability, disorder, and complexity. In ECG signal analysis, entropy has proven to be a valuable feature, particularly for diagnosing atrial fibrillation, one of the most prevalent arrhythmic disorders [39–41]. Our approach incorporates Shannon entropy, which measures the complexity of data and is defined as follows:

3 $$\begin{aligned} H(X) = - \sum _{i=1}^{n} p(x_i) \log _2 p(x_i), \end{aligned}$$

where * H(X) * represents the Shannon entropy of the * X *, $p(x_i)$ is the probability mass function of the * i *-th outcome $x_i$, $\log _2\,\,$ denotes the logarithm to the base 2, * n * is the number of possible outcomes for the random variable * X *, and $x_i$ represents the * i *-th outcome of the random variable. Shannon entropy was computed for each 5-s sample.

Additional features include heart rate, defined as the number of heartbeats per unit time, typically expressed in beats per minute (bpm). The heartbeat frequency corresponds to the cardiac cycle rate and is determined by the time intervals between consecutive peaks in the ECG signal. Another important feature is peak amplitude variability, quantified as the standard deviation of amplitude differences between successive peaks. Lastly, the average distance between peaks represents the mean elapsed time between consecutive heartbeats, providing insights into the cardiac rhythm’s behavior. These features are widely employed in cardiology research for diagnosing arrhythmic disorders and are straightforward to compute [1].

A more detailed description of similar features can be found in Frausto et al. [42], where the authors proposed a comparable feature vector to classify SERS spectra of organophosphate pesticides. Collectively, these 17 metrics form a comprehensive feature vector that captures relevant signal characteristics, enabling the neural network to maximize performance while maintaining low algorithmic complexity.

We advocate the use of these features to extract significant, easily computed descriptors that effectively characterize the ECG signal’s behavior and key morphological traits, thereby eliminating the need for deeper neural network architectures.

### Computational efficiency

To assess the suitability of the proposed model for deployment in portable medical devices and mobile health applications, we quantified its computational footprint. The network contains 154 trainable parameters, distributed across the batch-normalization layer (34), the five-neuron hidden layer (90), and the five-neuron softmax output layer (30); an additional 34 non-trainable parameters store the running statistics of the batch-normalization layer. On an Intel Core i7 CPU (no GPU acceleration), a complete training realization (220 epochs, batch size 20) requires 9.33 seconds on average, while the inference time for a single 5-second ECG segment is 1.014 ms. The feature-extraction stage adds 1.937 ms per sample, giving a total end-to-end latency of approximately 3 ms, well below the millisecond-scale budgets typical of embedded biosignal processors, making the pipeline a promising candidate for low-power deployment.

## Results

**Fig. 4:**
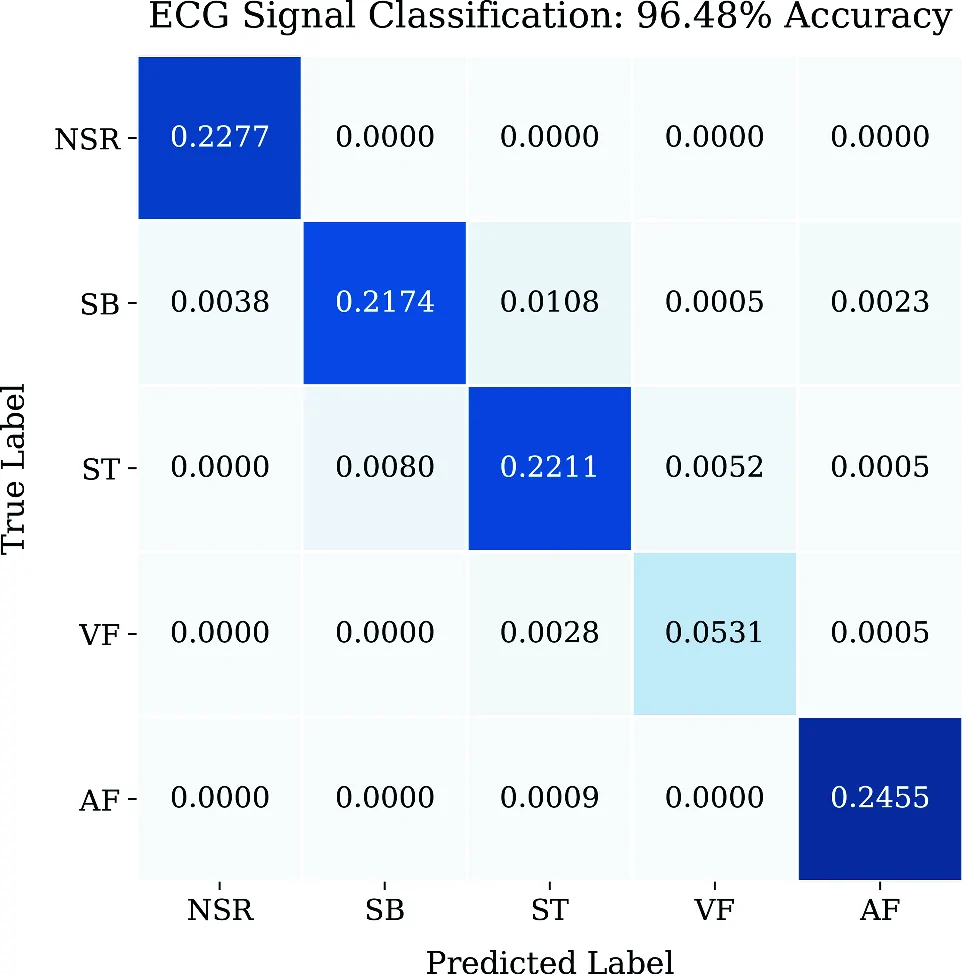
Aggregated confusion matrix of the compact ANN, computed across the 50 disjoint test folds of the stratified 10-fold cross-validation repeated 5 times. Cell values are global proportions ($n_{ij}/N$, with all cells summing to one); diagonal entries reflect both per-class accuracy and the relative prevalence of each class in the dataset

Predictions from the 50 disjoint test folds of the stratified cross-validation protocol described in Methodology section were aggregated to construct the global confusion matrix presented in Fig. 4. Our compact neural network algorithm achieved a mean accuracy of $96.48\% \pm 2.50\%$. To complement this figure and account for the class imbalance present in the dataset—particularly for the ventricular flutter class, which represents only *5.6%* of the samples—we additionally report Cohen’s kappa (*κ *) and the Matthews correlation coefficient (MCC), both of which are widely recommended for imbalanced multi-class classification tasks. Across all 50 folds, our model achieved $\kappa = 0.9546 \pm 0.0321$ and $\textrm{MCC} = 0.9557 \pm 0.0313$. Both values, well above the 0.80 threshold conventionally interpreted as “almost perfect agreement,” confirm that the reported accuracy is not an artifact of the majority classes but reflects genuine discrimination across all five categories. We additionally report a macro-averaged F1 score of *0.9586 ± 0.0347*, with macro-averaged precision of *0.9632 ± 0.0337* and recall of *0.9628 ± 0.0319*. These results indicate that the features extracted from the ECG signals provide a reliable, comprehensive and relevant data source for the detection and classification of arrhythmic disorders. Further analysis of the per-class performance is documented in Table 1.

**Table 1:** Performance metrics of the classification algorithm for each class

Class	Precision	Recall	F1 score
NSR	0.98	1.00	0.99
SB	0.96	0.93	0.94
ST	0.94	0.94	0.94
VF	0.90	0.94	0.92
AF	0.99	1.00	0.99

**Table 2:** Comparison of methods for arrhythmia classification. We note that the cited works differ in datasets, sampling rates, number of classes, and class definitions; the reported accuracies should therefore be interpreted as indicative of each method’s reported performance in its respective setting rather than as a strictly controlled head-to-head benchmark

Type of network	Acc. (%)	# Classes	Year	References	Dataset	Approx. params/depth	Evaluation protocol	Feature engineering	Arrhythmias considered
Compact ANN (ours)	96.48	5	This work	–	MIT-BIH + INCART	154 trainable params, 1 hidden layer	Stratified 10-fold CV, 5 repeats	17 engineered features	NSR, SB, ST, VF, AF
Wavelet + ANN	96.67	–	2017	[43]	MIT-BIH	Shallow ANN	Single split	Multiresolution wavelet	Multi-class (different panel)
Self-ONN (1-D)	82/ 94	2	2024	[44]	MIT-BIH	Compact (1-D ONN)	Train/test split	Feature injection	SVEP, VEP only
12-layer CNN	97.80	5	2024	[45]	MIT-BIH	*∼ *1M+ params	Train/test split	None (end-to-end)	LBBB, RBBB, APC, PVC, NSR
1-D CNN (64 filters)	98.63	4	2023	[10]	MIT-BIH	High (CNN, residual)	Train/test split	None	SVEP, VEP, fusion, NSR
2-D CNN	96.04	4	2018	[16]	MIT-BIH	Medium (2-D CNN)	Train/test split	None	SVEP, VEP, fusion, NSR
CNN	92.50	4	2017	[46]	MIT-BIH	Medium	Train/test split	None	ST, AF, AFL, NSR
Decision tree	96.30	4	2016	[47]	MIT-BIH	Tree-based	Train/test split	Nonlinear features	AF, AFL, VF, NSR

**Fig. 5:**
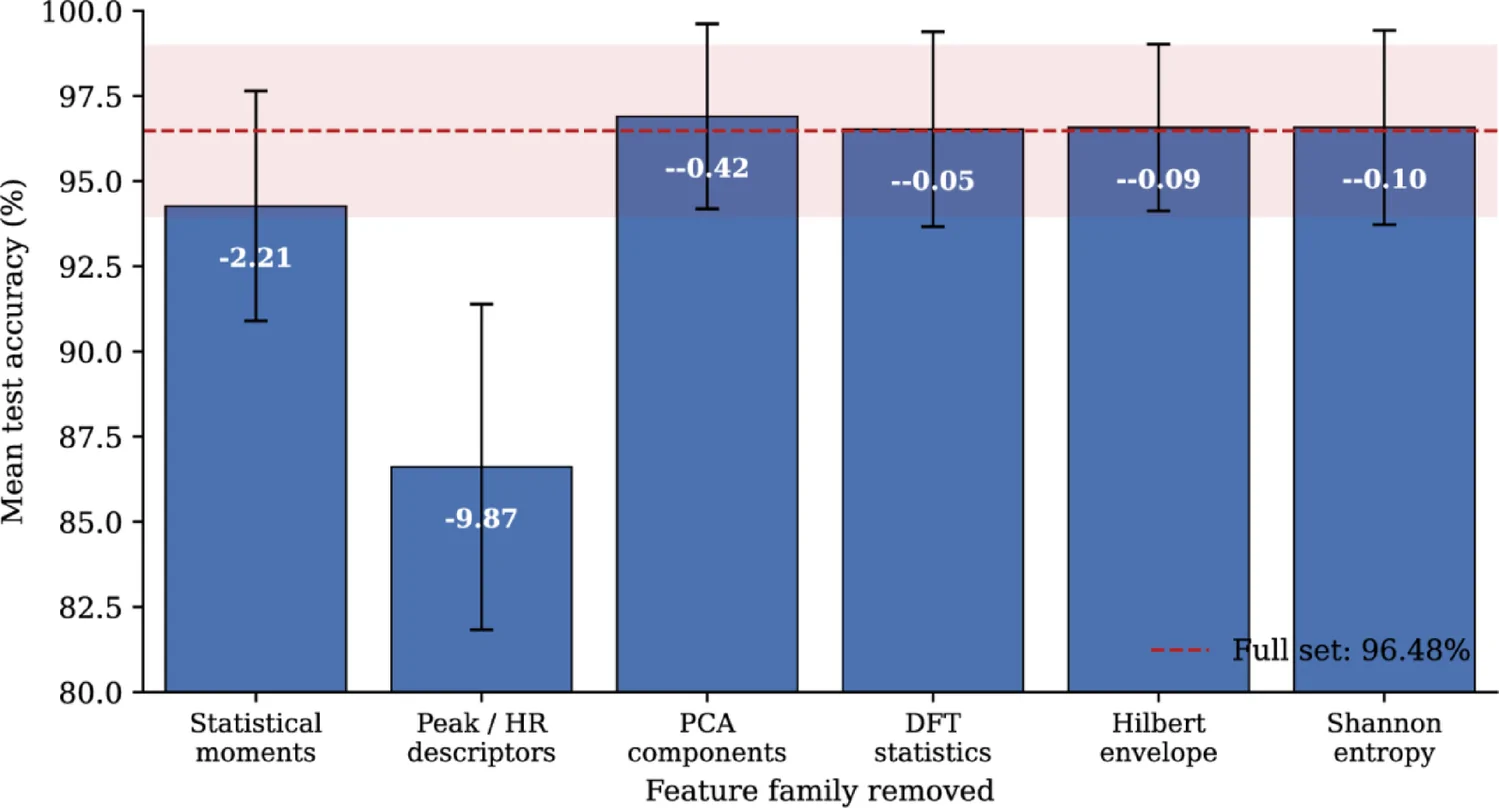
Feature-family ablation. Bars show the mean test accuracy of the compact ANN when one feature family is removed from the input vector, evaluated under the same stratified 10-fold cross-validation protocol used in the main experiment. The dashed line and shaded band indicate the mean and one-standard-deviation interval of the model trained on the full feature set. Numbers on the bars report the accuracy drop with respect to the full-feature baseline; larger drops indicate more informative families. Removing the peak/heart-rate descriptors causes the largest degradation (*∼ *10 percentage points), consistent with the rhythm-based clinical definition of the considered arrhythmias

The achieved performance of our model is comparable to that of other state-of-the-art models. To contextualize our results, we compare our approach with several relevant studies from the literature. Ref. [43] introduced a neural network incorporating wavelet transform-based engineered features, achieving an accuracy of 96.48%. In Ref. [44] developed a relatively compact and low computationally complex 1-D Self-Operational Neural Network (Self-ONN) for ECG classification. Nonetheless, this model was limited to classifying ectopic and ventricular ectopic beats, achieving 82% and 94% accuracy, respectively. On the other hand, Ref. [45] presented a complex 12-layer CNN for classifying arrhythmic disorders similar to those used in this study. The model achieved a slightly higher accuracy of 97.8%. Nevertheless, this performance came at the cost of a significantly more complex architecture, which increases computational complexity. In another approach, Ref. [10] implemented a 64-filter, 1-D CNN with a superior average accuracy of 98.63%. However, our algorithm offers a comparable performance with lower computational complexity, making it more suitable for real-world applications that often face resource-limited environments. In Ref. [46] presented a CNN model that classified similar disorders to those used in this study with an accuracy of 92.5%. In a separate study, [47] proposed a decision tree-based approach that achieved an accuracy of 96.3%. As demonstrated, our algorithm performs similarly to more advanced models, including CNN-based approaches. However, it achieves comparable accuracy while maintaining minimal computational complexity. A complete comparison of our model with other state-of-the-art algorithms is presented in Table 2. The relative contribution of each feature family to this performance is examined in the ablation study reported in Fig. 5, which quantifies the accuracy drop incurred when each of the six feature families is removed from the 17-dimensional descriptor. The peak/heart-rate descriptors emerge as the most informative family, followed by the statistical moments, while the PCA, DFT, Hilbert-envelope, and Shannon-entropy families contribute marginally once the dominant families are present. This result supports the design choice of a compact, heterogeneous feature vector rather than a single-family representation.

It is important to mention that the cited works in Table 2 employ different datasets, different class definitions, and heterogeneous evaluation protocols. However, two patterns clearly emerge. First, models with significantly higher parameter counts (e.g., the 12-layer CNN of Ref. [45] and the residual 1-D CNN of Ref. [10]) outperform our model by less than 1.3 percentage points in accuracy, while requiring at least four orders of magnitude more parameters. Second, classical and shallow approaches that match our parameter budget (Refs. [43, 47]) deliver accuracies between *96.3%* and *96.7%*, comparable to the *96.48%* reported here. This positions our compact ANN within a favorable region of the accuracy–efficiency trade-off curve.

## Discussion

Inspection of the confusion matrix in Fig. 4 reveals that NSR and AF are recovered with near-perfect recall, while VF (the most under-represented class) is now classified with a recall of 0.94, comparable to the larger classes. The principal source of residual confusion is between Sinus Bradycardia and Sinus Tachycardia, two rhythm classes that share morphological similarity outside their characteristic heart-rate ranges. The use of class-balanced weighting during training partially mitigates the imbalance of the VF class but does not fully eliminate inter-class confusion in the sinus rhythms; collecting additional samples from the borderline regimes (e.g. patients near the 60 bpm or 100 bpm boundaries) is the most direct route to further improvement. The feature-ablation analysis presented in Fig. 5 provides further insight into the model’s behaviour. The dominance of the peak/heart-rate descriptors (*∼ *10 percentage-point drop when removed) and the statistical moments (*∼ *2 percentage-point drop) is consistent with the rhythm-based clinical definition of the arrhythmias considered, which are primarily characterized by alterations of the heart rate and of the morphological regularity of consecutive beats. The remaining feature families (PCA, DFT, Hilbert envelope, and Shannon entropy) contribute marginally on their own but together provide the complementary spectral, time–frequency and statistical context that allows the five-neuron classifier to reach the reported performance. This decomposition supports the interpretation of the proposed descriptor as a heterogeneous, complementary representation rather than a redundant collection of features.

Beyond classification accuracy, a second dimension along which the proposed model should be evaluated is its computational footprint. As reported in Computational efficiency section, the compact ANN contains only 154 trainable parameters and runs end-to-end in approximately 3 ms per 5-s segment on a standard CPU. By comparison, the 12-layer CNN of Ref. [45] and the 1-D residual CNN of Ref. [10] contain on the order of $10^{5}$–$10^{6}$ trainable parameters, placing our pipeline well within the budgets of typical embedded platforms.

These favourable accuracy and efficiency results, however, must be interpreted in light of several limitations. First, the dataset is restricted to two PhysioNet sources and a single ECG lead (Lead I); generalization to multi-lead recordings, ambulatory data, and heterogeneous patient populations has not been evaluated. Second, the classifier was trained on 5-second segments containing a continuous arrhythmic pattern, which is a simplification of the noisy, transient events encountered in clinical practice. Third, the comparison reported in Table 2 is drawn from heterogeneous experimental settings: dataset, sampling rate, segment length, number and definition of classes, and evaluation protocol all vary across studies. Direct numerical comparison is therefore only indicative; we report these values to situate our work within the landscape of related approaches rather than to claim strict superiority. A controlled benchmark on a common dataset is left for future work.

Although the reported performance is encouraging, the model presented here should be regarded as a proof-of-concept prototype, not as a clinically validated diagnostic tool. Real-time deployment claims are based on inference latency on a desktop CPU and have not yet been validated on actual embedded hardware or in a clinical workflow. Prospective validation on independent multi-centre cohorts, evaluation under realistic noise conditions, and assessment of robustness to demographic and pathological diversity are necessary prerequisites for any clinical use.

## Conclusion

Our research makes a significant contribution to the study of arrhythmic disorders through ECG signal analysis and machine learning algorithms. We have demonstrated that compact neural networks offer a robust, ML-based approach for ECG classification tasks. Notably, the simplicity of the proposed neural network architecture enables straightforward software implementation, facilitating integration into diverse healthcare settings, including those with limited technological resources. Beyond the neural network model itself, the proposed feature engineering techniques allow us to extract relevant and intricate information from ECG signals. The performance of our model is comparable to that of state-of-the-art algorithms. We encourage further exploration of feature engineering as an effective strategy to develop simpler, more accessible, and robust machine learning solutions for arrhythmic disorder detection and classification.

## Data Availability

The source code for this study is available at https://github.com/CFATA-AI/ECG-CLASSIFICATION. The research utilized two PhysioNet databases MIT-BIH Arrhythmia Database (https://www.physionet.org/content/mitdb/1.0.0) and INCART DB (https://physionet.org/content/incartdb/1.0.0).
